# Gender differences in drug-related risks among underground rave attendees in Spain

**DOI:** 10.1192/j.eurpsy.2026.12048

**Published:** 2026-07-31

**Authors:** F. Calvo, R. Clua-García

**Affiliations:** 1 Departament de Pedagogia, Universitat de Girona, Girona; 2 Faculty of Health Sciences at Manresa, University of Vic, Vic, Spain

## Abstract

**Introduction:**

Rave parties are leisure contexts strongly associated with polysubstance use and high-risk behaviors. While previous research has addressed harm reduction and consumption patterns in nightlife, little quantitative evidence exists on gender differences in underground raves, settings characterized by high prevalence of stimulant and hallucinogenic use. Understanding gender-related risks is crucial to design effective harm reduction strategies.

**Objectives:**

To analyze gender differences in drug-related risks, binge drinking, and sexual behaviors among attendees of underground raves in Girona, Spain.

**Methods:**

A cross-sectional survey was distributed online and in rave contexts using snowball sampling (April 2023). The questionnaire, adapted from validated harm reduction instruments, assessed sociodemographic data, substance use (alcohol, cannabis, stimulants, psychedelics, depressants), binge drinking, and drug-related and sexual risk behaviors. Descriptive statistics, chi-square tests, and binary logistic regressions were conducted. Ethical approval and informed consent were obtained.

**Results:**

A total of 253 participants (mean age 23.4 years; 51.4% women, 41.5% men, 7.1% other genders) were included. Almost all reported lifetime drug use: alcohol (94.9%), cannabis (85.5%), MDMA (71.4%), speed (64.7%), cocaine (56.1%), ketamine (43.9%). Binge drinking was reported by 78.0%, with 48.2% in the last 30 days. The most prevalent risk behaviors were polysubstance use in the same session (71.8%), public consumption (65.5%), and lack of substance testing (56.5%). Regarding sexual risk, 62% had intercourse under drug effects, 43.9% without protection, 20.2% with strangers, and 3.5% reported sexual assault. Gender differences showed that women were more likely than men to report increased receptivity to sex (60.0% vs. 38.1%) and intercourse under drug influence (70.0% vs. 50.5%). Logistic regressions confirmed female gender as a predictor of higher sexual risk behaviors (OR 2.08; CI95% 1.30–3.35). Women were also more likely to consume substances despite health problems (OR 1.74; CI95% 1.03–2.92). No significant gender differences were observed in binge drinking prevalence.

**Conclusions:**

Underground raves constitute high-risk environments marked by intense polysubstance use and binge drinking. Gender differences are evident, with women reporting more sexual risk behaviors and drug use despite health issues. These findings highlight the need for gender-sensitive harm reduction interventions, including drug checking services, safer sex education, and preventive strategies targeting rave organizers and attendees.

**Disclosure of Interest:**

None Declared

